# Unexpected perturbations training improves balance control and voluntary stepping times in older adults - a double blind randomized control trial

**DOI:** 10.1186/s12877-016-0223-4

**Published:** 2016-03-04

**Authors:** Ilan Kurz, Yoav Gimmon, Amir Shapiro, Ronen Debi, Yoram Snir, Itshak Melzer

**Affiliations:** Schwartz Movement Analysis & Rehabilitation Laboratory, Department of Physical Therapy, Recanati School for Community Health Professions, Faculty of Health Sciences, Ben-Gurion University of the Negev, P.O.B. 653, Beer-Sheva, 84105 Israel; Department of Mechanical Engineering, Faculty of Engineering, Ben-Gurion University of the Negev, Beer-Sheva, Israel; Orthopedic department, Barzilai Medical Center, Ashkelon, Israel; “Beit Yona” senior citizens home, Beer-Sheva, Israel

**Keywords:** Aging, Falls, Unexpected perturbation of balance, Step execution, Postural stability

## Abstract

**Background:**

Falls are common among elderly, most of them occur while slipping or tripping during walking. We aimed to explore whether a training program that incorporates unexpected loss of balance during walking able to improve risk factors for falls.

**Methods:**

In a double-blind randomized controlled trial 53 community dwelling older adults (age 80.1±5.6 years), were recruited and randomly allocated to an intervention group (*n* = 27) or a control group (*n* = 26). The intervention group received 24 training sessions over 3 months that included unexpected perturbation of balance exercises during treadmill walking. The control group performed treadmill walking with no perturbations. The primary outcome measures were the voluntary step execution times, traditional postural sway parameters and Stabilogram-Diffusion Analysis. The secondary outcome measures were the fall efficacy Scale (FES), self-reported late life function (LLFDI), and Performance-Oriented Mobility Assessment (POMA).

**Results:**

Compared to control, participation in intervention program that includes unexpected loss of balance during walking led to faster Voluntary Step Execution Times under single (*p* = 0.002; effect size [ES] =0.75) and dual task (*p* = 0.003; [ES] = 0.89) conditions; intervention group subjects showed improvement in Short-term Effective diffusion coefficients in the mediolateral direction of the Stabilogram-Diffusion Analysis under eyes closed conditions (*p* = 0.012, [ES] = 0.92). Compared to control there were no significant changes in FES, LLFDI, and POMA.

**Conclusions:**

An intervention program that includes unexpected loss of balance during walking can improve voluntary stepping times and balance control, both previously reported as risk factors for falls. This however, did not transferred to a change self-reported function and FES.

**Trial registration:**

ClinicalTrials.gov Registration number: NCT01439451.

## Background

Falls are a major problem among elderly population; they are the leading cause of injury above the age of 65 [1]. Of those who fall in the U.S., 20 to 30 % suffer moderate to severe injuries that reduce mobility and independence, and increase the risk of death [2]. The financial impact in 2000, adjusted for inflation, was $30 billion and is expected to reach $67.7 billion by 2020 [1].

Walking is the major activity in which large proportion of falls in older adults occurs [3]. Sixty percent of outdoor falls among older adults resulted from slips or trips [4]. Even among older adults capable of independent walking, there could be a substantial decline in balance performance, which does not become evident until a slip or a trip happens [5]. In fact, the inability to step rapidly in response to unexpected loss of balance ultimately determines whether a fall occurs [6, 7]. Thus, a better way to improve balance, improve stepping and reduce risk of falls may be to direct preventive efforts towards older adults who have not yet fallen. Until recently these balance recovery responses were considered hardwired postural reflexes that could not be influenced by training. However, [8–10] it was showed that older adults were able to adapt in a reactive manner after participation in a perturbation exercises that challenged the mechanisms responsible for dynamic stability (i.e., increase in base of support and counter-rotating segments around the center of mass).

A number of studies have begun to examine the effect of perturbation training on balance of older adults. Shimada et al. [11] found improvement in mobility and a trend to fall reduction after split treadmill training. This training method is a very unnatural condition, given that most people walk with the same velocity in each leg. Other studies have other issues. Pai et al. [12] showed a rapid decrease in loss of balance in response to multiple presentations of a slip perturbation after rising from sit to stand. Mansfield et al [13] found that older adults with a history of falls or instability reduced the frequency of multi-step reactions and foot collisions after perturbation training while standing or walking in place. The training methods perturb the balance of their participants from sit to stand or during standing or walking in place, which may not be as relevant in a natural setting as might be a perturbation while walking. Melzer and Oddsson [14] found improvement in voluntary stepping, and balance control, in an exercises program that incorporate mild external balance perturbation exercises applied by the instructors; and Halvarsson et al. [15] found that old fallers that suffered from fear of falling, decreased their fear of falling, and voluntary stepping times during dual-task performance and increased velocity of walking post perturbation training.. However, in these program the perturbations of posture were expected, and not random. Recently it was showed that unidirectional translational treadmill training (i.e., a laboratory-induced trip) reduced falls [16, 17]. Bhatt et al. [18] found that inducing unannounced right-leg slips, participants significantly reduced fall and balance loss incidence. Pai, et al. [19] found that a single session of repeated-slip exposure reduced older adults’ annual risk of falls from 34 to 15 % (*p* < 0.05) especially among those who had history of falls. The above protocol provided an anterior perturbation, causing a backwards “slip” initiated always by on the right foot. Participants might have learned and expected the right-leg slips perturbations. In a recent meta-analysis [20] that include 8 perturbation-based balance training studies (*n* = 404) participants reported fewer falls than those in the control groups.

Motivated by the above perturbations training studies and trying to accommodate for some of the issues mentioned above (i.e., perturbation training while standing or walking in place; highly predictable repeated-right leg slip exposure; a very unnatural walking on split treadmill), we aimed to explore whether unexpected multidirectional perturbation training while walking on a treadmill [21] can reduce risks of falls in independent older adults. A perturbation exercise while walking provides a more realistic balance training that is sufficiently task-specific so that responses on this training regime will be more likely to be transferred to other measures of balance control and voluntary stepping measures.

Our hypotheses were that following exposure to a gait training program that includes unexpected perturbations exercises during walking will significantly improve voluntary stepping times as well as balance control in older adults, two factors that are associated with falls and injuries related with falls [22–27]. We believe that perturbation exercises that will challenge both balance control during walking as well as trigger a quick stepping responses to avoid fall during walking. These postural response following an external perturbation receives a higher priority than a voluntary action thus can be incorporated into centrally programmed voluntary movements [28]. This concept should be of importance for balance training and it further supports the notion that postural perturbations should be incorporated into balance training programs.

## Methods

### Participants

**Fig. 1:**
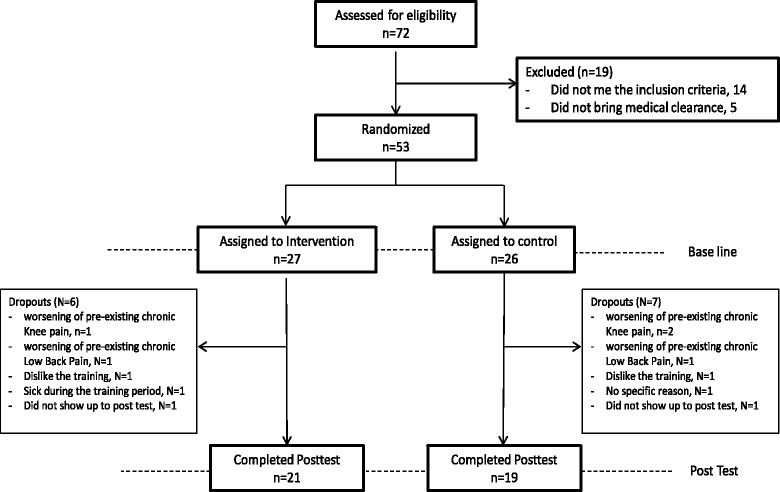
Study flow chart

Community dwelling older adults were recruited from two protected housing institutes. Eligibility criteria were: 70 years or older; walking independently; Mini-Mental Score higher than 24; no severe focal muscle weakness or blindness; no known neurological disorders; no metastatic cancer. Out of 72 seniors who were assessed for eligibility, 19 were excluded (see Fig. 1). All subjects provided a medical waiver signed by their primary care physician clearing them to participate in moderate physical exercise. The study was approved by the Helsinki committee of Barzilai University medical center, Ashkelon, Israel (ClinicalTrials.gov Registration number #NCT01439451). All subjects signed an informed consent statement.

### Study design

After eligibility and baseline assessments subjects were randomized to two blocks (27 and 26 subjects, respectively). In the first site, 28 subjects were randomly allocated to 2 intervention groups and in the second site 25 subjects were randomly allocated to 2 intervention groups. The subjects random allocation was made by an investigator not involved in the assessments using computer random allocation software (Random allocation software version 1.1, Isfahan Iran). Performance-based and laboratory balance functions were tested before and after the training period by a blinded investigator. All assessment sessions were performed at the same time of day, and in the same order.

### Training programs

**Fig. 2:**
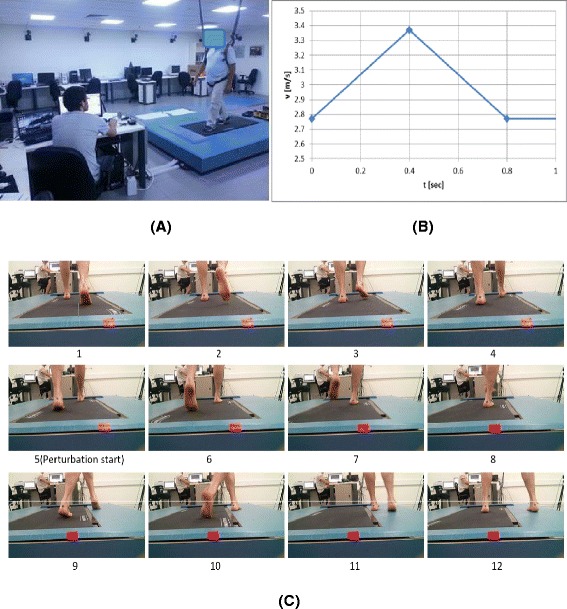
The perturbation treadmill system used **a** Photo of the perturbation system during balance training. The system is compose of a motor-driven treadmill, mounted on a moving platform, motion controller, safety harness and an operator station; **b** the perturbations velocity control diagram during training delivered unpredictably in forward, backward, left, and right directions. Note those are actual measurements taken during perturbation training. **c** Example of the perturbation applied during the treadmill walking training (*c1*–*c12*). The perturbation applied unpredictably (*c5*) by horizontal movement of the platform towards the left side during the right foot initial contact-loading response phases of gait cycle. The participant right foot was slipped unpredictably to the left while walking in the center of the platform. The participants performed a cross over stepping response by his left foot (*c6*–*c9*), than additional side step was performed by the right foot stepping outside the treadmill (*c10*–*c12*)

We used a mechatronic device that provides controlled and unexpected anterior-posterior and Medio-lateral platform translations during a single belt, treadmill walking (details in reference [21] and Fig. 2a-c). The intervention group received 24 training sessions, twice a week for 12 weeks. Each session lasted for about 20 min and included 3 min warm-up walking in subject own preferred pace, 14 min of unannounced perturbations exercises, given in random direction order, during walking (every 20–40 s) and 3 min of cool down walking. During the training sessions the subjects were instructed to walk on a treadmill, wearing their own walking shoes, with their hands free to swing; there were no handrails on the treadmill. To prevent injury if loss of balance occurred during the treadmill walking, the subject wore a loose safety harness that could arrest the fall, but that allowed the subject to walk comfortably as well as freedom to execute recovery reactions without suspension (Fig. 2a). The instructions given to the subjects were: “Walk as naturally as possible at your preferred stride frequency”. The treadmill’s walking speed was adjusted to the subjects own preferred speed.

The perturbations were in anterior-posterior direction (i.e., sudden acceleration or stop of treadmills belt) and sudden medio-lateral horizontal translation of the treadmill that challenges the medio-lateral dynamic control. During all sessions, 400 ms horizontal surface translations were delivered as the subject walked on the treadmill. The velocity profiles were triangular waveforms with peak velocities of 0.1–3.2 m/s, resulting in displacements of 1–18 cm and peak accelerations of 0.5–16.0 m/s^2^. Perturbation timing was preset and therefore was not given in a specific phase of the gait cycle or to a specific leg. The perturbation training program had 24 levels of difficulty with increasing levels of perturbations (i.e., increased displacement, velocity and accelerations of the horizontal translations, see Table 1). The difficulty level was adjusted according to the subject abilities, starting from the lowest level of 1 cm displacement at 0.1 m/s velocity and 0.5 m/s^2^ acceleration at the first training session. If the subject was able to recover from all perturbations during the session (i.e., did not fell during the session) and felt that he can be further challenged, a higher level of perturbation was introduced in the next session. If not, the same level of perturbation was introduced again until successfully dealt with. Fall during the training session was defined as load cell sensors detected 30 % or more body weight suspended by the safety harness.

**Table 1:** Details of Protocol Used in the perturbation intervention training program

The training sessions #	platform displacement (cm)	platform peak velocity (cm/s)	platform peak acceleration (cm/s^2^)	Number of unannounced random Perturbations per minute (forward, backward, left, right)
1	1–2 cm	0.1–0.5 m/s	0.5–3.0 m/s^2^	1
2	2–3 cm	0.2–0.6 m/s	0.7–5.0 m/s^2^	2
3	3–4 cm	0.4–0.6 m/s	0.9–7.0 m/s^2^	1
4	3–5 cm	0.5–0.7 m/s	1.1–7.0 m/s^2^	2
5	4–6 cm	0.5–0.8 m/s	1.5–8.0 m/s^2^	2
6	5–6 cm	0.5–1.0 m/s	2.0–10.0 m/s^2^	2
7	5–7 cm	0.7–1.0 m/s	2.5–12.0 m/s^2^	2
8	6–7 cm	0.8–1.2 m/s	3.0–14.0 m/s^2^	3
9	6–8 cm	1.0–1.5 m/s	3.5–16.0 m/s^2^	2
10	7–8 cm	1.2–1.8 m/s	4.0–16.0 m/s^2^	3
11	7–9 cm	1.5–2.0 m/s	5.0–16.0 m/s^2^	2
12	8–9 cm	1.6–2.2 m/s	6.0–16.0 m/s^2^	3
13	8–10 cm	1.8–2.5 m/s	7.0–16.0 m/s^2^	2
14	9–10 cm	2.0–2.6 m/s	8.0–16.0 m/s^2^	3
15	9–11 cm	2.0–2.8 m/s	9.0–16.0 m/s^2^	2
16	10–11 cm	2.2–3.0 m/s	10.0–16.0 m/s^2^	3
17	11–14 cm	2.4–3.0 m/s	11.0–16.0 m/s^2^	2
18	12–14 cm	2.5–3.0 m/s	12.0–16.0 m/s^2^	3
19	13–15 cm	2.6–3.0 m/s	13.0–16.0 m/s^2^	2
20	14–15 cm	2.6–3.2 m/s	14.0–16.0 m/s^2^	3
21	14–16 cm	2.8–3.2 m/s	14.0–16.0 m/s^2^	2
22	15–17 cm	2.8–3.2 m/s	14.0–16.0 m/s^2^	3
23	16–18 cm	2.8–3.2 m/s	14.0–16.0 m/s^2^	2
24	17–18 cm	2.8–3.2 m/s	14.0–16.0 m/s^2^	3

The 24 training sessions. Each session lasted 20 min, included 3 min warm-up treadmill walking, 14 min of perturbations during comfortable treadmill walking, given in random direction (right, left, forward and backwards), and 3 min of cool down walking. The perturbation training program had 24 levels of difficulty with increasing levels of perturbations (i.e., increased displacement, velocity and accelerations of the horizontal translations). During each session, the listed platform translation unannounced perturbations were delivered in an unpredictable randomized sequence, in the directions indicated (forward, backward, left, and right). Perturbations for the treadmill walking were occur randomly (i.e., occurs in all phases of gait cycle) in order to increase the ecological validity. The perturbation was delivered after 20–30 s approximately every 20 strides and was triggered randomly

The control group received 24 sessions, twice a week for 12 weeks, 20 min treadmill walking on the same treadmill but without unexpected perturbations. similar to the intervention group the control group subjects walked at their own preferred speed and in their own walking shoes, with their hands free to swing; there were no handrails on the treadmill, thus they wore a loose safety harnesses that allowed comfortable walking. Since both group trained on the same system, they were blinded to the allocation to intervention or control group.

### Assessments

For the balance control testing the subjects were instructed to stand barefoot as still as possible and on a force platform in a standardized stance, their feet close together. Ten 30-s quiet-standing trials with eyes blindfolded. Center of pressure and ground reaction force data were collected with a Kistler 9287 force platform (Kistler Instrument Corp, Amherst, NY, USA), sampled at a frequency of 100 Hz. Evaluation of balance control was made using both traditional measure of postural sway in eyes closed condition (e.g. ML-sway, AP-sway, mean sway velocity, and Mean sway area), we also calculated the Stabilogram-Diffusion Analysis parameters from Center of pressure data. The Stabilogram-Diffusion Analysis plots (SDA) of the mean square center of pressure displacement (Critical Displacement, Cd) vs. time interval (Critical Time, Ct) parameters were extracted from the center of pressure trajectories. The SDA plots derived from COP trajectories during standing indicate the presence of two different behaviors depending on the time interval of interest. For shorter time intervals (less than 1 s) the COP tend to drift away from a relative equilibrium point while longer time intervals (more than 1 s) the COP tends to return to a relative equilibrium point [29]. It has been suggested that long-term region is governed by closed-loop control mechanisms whereas the postural control systems operate with sensory feedback, while during the short-term region the postural control system is governed by open-loop control mechanisms whereas the postural control systems operate without sensory feedback. The transition point between the short-term and long-term behavior has been termed the Critical Time (Ct) and sway displacement has been termed the Critical Displacement (Cd) at which closed-loop control begins to dominate sway behavior. It was described in detail by Collins and De Luca [29, 30]. The SDA method has been adopted by our research group, we found that SDA parameters (e.g., Critical Displacement (*Cd*), and Short-term Effective diffusion coefficients (*Ds*) were able to predict falls [26] and injury from fall [27].

For the Voluntary Step Execution Test participants were instructed to stand with both feet on a single force platform, they were instructed to voluntary step as quickly as possible following a somatosensory cue, given randomly on one of their feet [24, 31, 32]. Center of pressure movement and ground reaction force data were collected from the force platform, sampled at a frequency of 100 Hz. A total of 8 trials were conducted in single task condition, 4 forward and 4 backward as well as in dual task conditions. The average result across task condition was used for statistical analysis. For the single task, subjects viewed an “**X**” displayed on a screen in front of them. During the dual task they conducted the same test while performing the modified Stroop task [33, 34]. Specific temporal events were extracted from the step execution data: (a) Reaction Time; (b) Foot Contact Time; (c) Preparation Time; (d) Swing Time; as previously described in detail [24, 31, 32]. The foot contact times (i.e., stepping time) and reaction time duration especially in dual task condition were able to predict future fall [23] and injury from fall [22], thus both were selected as the primary outcome measure in the present study.

The secondary outcome measures were the self-reported function (Late Life Function and Disability Instrument (LLFDI)) [35], Fall Efficacy Scale (FES) [36] and performed the Performance-Oriented Mobility Assessment (POMA) [37].

### Sample size

Sample size requirements were calculated based on AP postural sway in eyes closed condition and voluntary step execution times, both were found to predict injury from fall in older adults ([22, 27] respectively). For both calculations, the probability of type I error was 0.05, and probability of type II error was 0.2. Based on data presented by Kurz et al. [27] that found that the traditional AP postural sway in eyes closed condition was 42.3 mm in older adults who were injured as a result of falling compared with 32.6 mm in non-fallers older adults; and Melzer et al. [22] found that the step execution times (i.e., foot contact time) in dual task condition of older adults who fell and as a result injured were 217 ms longer than those of non-fallers (1,394 ms vs. 1,177 ms). Using net reduction values (9.7 mm and 217 ms, respectively) in combination with the initial variance estimates (standard deviations of 11 mm and 250 ms, respectively), it was determined that 21 and 22 participants per group would be required, respectively. To account for reported attrition rates of about 25 % in studies involving older adults [38], we decided to include about 27 participants in each group for a total of 54 (22 × 1.25 = 27).

### Data and statistical analysis

PASW Statistics version 18.0 was used for statistical calculations (Somers, NY, USA, version 18). Baseline characteristics were compared using Independent *t*-test and Mann–Whitney U-tests for continuous and ordinal variables, respectively. To analyze the effect of the intervention program a two-way repeated-measures ANOVA for within subjects (pre vs. post-test) and between group (perturbation intervention vs. control group) was performed. Since age was significantly different between intervention and control group subjects, we included age as a covariate in the analyses. The primary outcome variables were the parameters that we previously found to be related to falls and injury from fall: the step execution times in single and dual task conditions (i.e., foot contact-time) and step reaction time, traditional postural sway in eyes closed condition (ML- and AP- sway, sway velocity and mean sway area) as well as Stabilogram diffusion Analysis parameters (Critical Displacement (*Cd*), and Short-term Effective diffusion coefficients (*Ds*) in eyes closed condition. The secondary outcome measures were LLFDI, FES and POMA. An intention to treat analysis was conducted by carrying the last obtained measurements forward for those subjects who did not complete all aspects of the study. Adjustment of level of significance for multiple comparisons were made. For each testing procedure (e.g., single task voluntary stepping, dual task voluntary stepping, postural sway and SDA), a full Bonferroni correction was used to achieve an overall significance level of 0.05.

For the significant improvement the Effect Size (ES) of Hedge’s *g* was calculated. The ES of *g* was calculated by taking the difference between the means of both groups divided by the average population standard deviation (SD). To estimate the SD for *g*, baseline estimated SDs of both groups was pooled. When interpreting correlation magnitudes: 0.0–0.2 is considered small, 0.2–0.5 is considered moderate and 0.5–0.8 is considered large [39].

## Results

Other than age and height there were no significant differences between the groups at baseline (Table 2). During the training period we had 6 drop outs in the intervention group and 7 in the control group (Fig. 1).

**Table 2:** Baseline characteristics of intervention and reference group subjects: descriptive statistics and group comparisons. Values are means ±SD (95 % confidence interval for means)

	Intervention Group (*N* = 27)	Control Group (*N* = 26)	*p*-value
Age (year)	78.2 ± 5.6	81.4 ± 4.3	0.05
% Female	62 %	79 %	0.25
Number drugs/day	3.3 ± 1.7	4.2 ± 2.4	0.18
Height (cm)	161.5 ± 10.9	154.9 ± 6.9	0.03
Weight (Kg)	70.9 ± 14.9	65.5 ± 12.9	0.23
BMI (Kg /m^2^)	27.1 ± 4.4	27.8 ± 5.1	0.64
Mini-Mental State Examination	29.1 ± 1.4	28.7 ± 1.4	0.49
Fall efficacy scale	20.5 ± 4.3	22.8 ± 10.3	0.36
POMA score	14.8 ± 1.3	14.7 ± 1.4	0.87
Late life function			
- Overall Function	66.8 ± 9.6	66.5 ± 6.7	0.90
- Upper Extremity Function	82.9 ± 11.7	79.2 ± 8.0	0.26
- Basic Lower Extremity Function	82.4 ± 12.4	81.3 ± 13.6	0.79
- Advanced Lower Extremity Function	59.6 ± 12.4	61.2 ± 10.4	0.66

Note: *p*-value compares baselines means in the two groups and, unless otherwise indicated, are based on *t*-test or chi-square. * *P*-value based on Wilcoxon signed rank test and Mann–Whitney *U* test. Abbreviations: *cm* centimeters, *Kg* Kilograms, *Kg/m* ^*2*^ kg per meter squared

Table 3 show that the perturbation training resulted in a significant group-by-time decrease in AP-sway (*p* = 0.012, [ES] = 0.59). There were also a trend towards a group-by-time decrease with a large effect size in sway velocity, sway area and ML-sway post-training (*p* = 0. 115, [ES] =0.72; *p* = 0.119, [ES] =0.74; and *p* = 0.142, [ES] = 0.58, respectively); although these differences were not statistically significant, the effect size is considered to be moderate. The Stabilogram-Diffusion analysis in eyes closed condition showed a significant group-by-time decrease in *Dxs*, (*p* = 0.012, [ES] =0.78) and a trend towards significance in C*dy* and *Dys* (*p* = 0.028, [ES] = 0.92; and *p* = 0.095, [ES] =0.65, respectively) (Table 4).

**Table 3:** The effect of balance training on traditional sway Parameters under eyes closed condition. Values are means±1 SD (95 % confidence interval for means). A full Bonferroni correction (α-level 0.05/4 = 0.0125) was used for each of the four tests to achieve an overall significance level of 0.05

	Group	Baseline	post-test	ANOVA (Baseline to post-test) T	ANOVA (Baseline to post-test) T x G
ML-sway (mm)	Experimental	47.9 ± 15.3	44.6 ± 15.5	F = 0.215	F = 2.247
	Control	41.4 ± 8.8	40.5 ± 8.6	*p* = 0.792	*p* = 0.142
AP-sway (mm)	Experimental	38.6 ± 13	35.8 ± 10	F = 0.694	F = 5.315
	Control	33.8 ± 8	33.9 ± 7	*p* = 0. 711	*p* = 0.012
Velocity(mm^2^/sec)	Experimental	37.7 ± 12	34.2 ± 10	F = 0.086	F = 2.609
	Control	35.1 ± 8	35.0 ± 9	*p* = 0.770	*p* = 0.115
Sway Area (mm^2^)	Experimental	169.3 ± 9	148 ± 76	F = 0.056	F = 2.549
	Control	136.6 ± 48	135 ± 5	*p* = 0.815	*p* = 0.119

Note: Comparison of baseline and post-intervention between the two groups based on repeated measures ANOVA (Test × Group). Abbreviations: *G* group, *T* time, *mm* millimeters, *s* seconds

**Table 4:** The effect of balance training on Stabilogram Diffusion Parameters in eyes closed condition. Values are means±1 SD (95 % confidence interval for means). A full Bonferroni correction (α-level 0.05/4 = 0.0125) was used for each of the four tests during the two phases to achieve an overall significance level of 0.05

	Group	Baseline	post-test	ANOVA (Baseline to post-test) T	ANOVA (Baseline to post-test) T x G
Short-term Effective diffusion coefficients in mm^2^ s ^-1^ (*Dxs*)	Experimental	97.9 ± 71.3	84.7 ± 58.6	F = 0.856	F = 5.822
	Control	79.18 ± 38.2	87.4 ± 48.8	*p* = 0.391	*p* = 0.012
Short-term Effective diffusion coefficients in mm^2^ s ^-1^ (*Dys*)	Experimental	62.9 ± 54.8	47.3 ± 34.6	F = 0.009	F = 2.928
	Control	37.2 ± 29.4	38.4 ± 27.5	*p* = 0.925	*p* = 0.095
Critical (Mean-Squared) Displacement in mm^2^ (*Cdx*)	Experimental	131.2 ± 105	112 ± 82.7	F = 0.038	F = 1.916
	Control	98.9 ± 48.7	96.6 ± 42.7	*p* = 0.847	*p* = 0.157
Critical (Mean-Squared) Displacement in mm^2^ (*Cdy*)	Experimental	90.3 ± 72	69.0 ± 52.7	F = 1.360	F = 5.266
	Control	62.7 ± 30	65.9 ± 30.4	*p* = 0.251	*p* = 0.028

Note: Comparison of baseline and post-intervention between the two groups based on repeated measures ANOVA (Test × Group). Abbreviations: *G* group, *T* time, *mm* millimeters, *sec* seconds

Table 5 shows that the perturbation training resulted a significant group-by-time interaction for foot contact time of the voluntary step execution in both the single and dual task conditions (*p* = 0.002, effect size [ES] = 0.75 and *p* = 0.003 effect size [ES] = 0.89 respectively). In addition, a significant group-by-time interaction for the voluntary step reaction time in dual task condition (*p* = 0.010), and a trend towards a significant group-by-time interaction for single task condition (*p* = 0.057).

**Table 5:** Voluntary Step Execution Test times and the preparation phase times during single task and dual task conditions (mean ± SD). Values are means ± SD (95 % confidence interval for means). A full Bonferroni correction (α-level 0.05/2 = 0.025) was used for each of the two different task conditions (single and dual task condition) to achieve an overall significance level of 0.05

	Group	Baseline	post-test	ANOVA (Baseline to post-test) T	ANOVA (Baseline to post-test) T x G
Single task condition					
Reaction Time (ms)	Intervention	215 ± 40	194 ± 36	F = 0.002	F = 2.187
	control	219 ± 70	206 ± 57	*p* = 0.968	*p* = 0.057
Foot Contact Time (ms)	Intervention	1065 ± 16	993 ± 138	F = 0.474	F = 11.325
	control	1027 ± 147	1010 ± 143	*p* = 0.495	*p* = 0.002
Dual Task condition					
Reaction Time (ms)	Intervention	412 ± 174	346 ± 99	F = 1.881	F = 7.322
	control	354 ± 98	339 ± 100	*p* = 0.179	*p* = 0.010
Foot Contact Time (ms)	Intervention	1355 ± 243	1224 ± 172	F = 0.439	F = 9.857
	control	1250 ± 165	1240 ± 171	*p* = 0.512	*p* = 0.003

Note: Comparison of baseline and post-intervention between the two groups based on repeated measures ANOVA (Test × Group). Abbreviations: *G* group, *T* time, *ms* milliseconds

We found no significant group-by-time interaction effect for all components of the LLFDI as well as for FES and POMA.

With respect to side effects and adverse events, during the exercise training program, muscle soreness was experienced by some subjects, especially in the early stage of the training. Those effects were managed by adjusting the training intensity and the symptoms disappeared during training.

## Discussion

The results support in part our main hypotheses, unexpected perturbations training while walking can improve the ability to voluntarily step rapidly and standing balance control in older adults. These parameters have been shown in the past to predict injury from falls [19, 24]. This shows that the benefits of unexpected perturbation during walking were generalized to other aspects of balance. Our findings are supported by Pai and Bahtt [40] that suggested that the central nervous system makes adaptive improvements in proactive and reactive control of stability as a result of trial and error perturbation practice (i.e., a forward slip). They suggested that the central nervous system probably decreases the reliance on feedback corrective mechanisms for successful recovery and builds an internal representations to improve its feedforward control while walking. Pai and Bahtt training [40] used forward slip practice, the training program, while in the present study the direction of the perturbation was highly unpredictable (forward, backward, right and left perturbations), the intervention group subjects were unable to predict the direction of perturbation during the training. Thus it is unlikely that the improvement was due to feedforward control. Our results support the notion that the central nervous system probably increased the reliance on feedback corrective mechanisms for successful recovery. This is supported by the results of the SDA.

The significant improvement in the intervention group and the large effect size in the SDF parameters (ES = 0.65–0.92) in eyes closed conditions are promising. Age-related decrease in SDF parameters are well documented [30, 33] and indicates that a COP tends to drift away from the equilibrium point is a predictor to falls in elderly persons [26, 27, 29, 30, 41–43]. The improvement in the *Dxs* and *Cdy* as well as tendency towards improvement in *Dys* of SDA in eyes closed condition showed in this study indicates that the deterioration of balance control could be reversed by perturbation balance training. AP balance control in eyes close condition (*Dys* and *Cdy*) were found to be an important risk indicator of falls and injurious falls [26, 27]. Laughton et al. [41] found a greater *Dys* in elderly fallers compared with the young’s, and greater AP and ML sway in older adults who demonstrated lower scores in the POMA. Kurz et al. [27] found that a deterioration of AP postural control present higher risk of serious injury. Most Studies usually used traditional balance measures, which provide descriptive information on the postural sway, however it is very difficult to understand the mechanism of postural control using traditional COP statistics, this we performed SDA. We found training effects on open- and close-loop control mechanism when vision was occluded as indicted by a significant decrease in Critical Displacement in AP direction (*Cdy*). . Improvements in and Short-term Effective diffusion coefficients in ML direction (*Dxs*) as well as a trend towards a decreased in Short-term Effective diffusion coefficients in AP direction (*Dys*) further support this notion. These parameters reflect the degree of sway in the short term region. A decreased tendency to continue sway in an ongoing direction (Table 4) indicates a behavior that reflects a more stable balance control system. This indicates that the intervention group was able to better detect COP movement under their feet and initiate a more effectively close loop balance control. These suggest that the improved closed-loop balance control when vision was occluded would likely be from proprioceptive and/or maybe vestibular sources.

The improvement in step execution in the intervention group was seen during the step execution times i.e., foot contact times, in both ST and DT (72 ms in ST and 131 ms in DT, see Table 5). A shorter step execution indicates improvement in the ability to prevent a fall if balance is lost, consequently, the risk of fall and injury maybe reduced [22–24]. The improvement in the step execution in the intervention group was accompanied with improvement in step reaction times, in particular under dual task conditions. The step reaction time duration is mainly dependent on sensory detection thresholds, nerve conduction velocities and central neural processing times. A shortened step reaction time in dual task condition suggests that the central neural processing time was improved as a result of perturbation training. This indicate that the executive functioning was improved, subject were able to step quickly while their attention was allocated elsewhere. The executive functioning is related to the ability to rapidly shift attention from a cognitive task to the stepping task. This could be interpreted also in terms of automaticity of the stepping behavior as an essential characteristic of the central reorganization process. Therefore, we could assume that the interference effect during dual task stepping was reduced as a result of the treadmill perturbation training.

Halvarsson et al. [15] also found significant improvements in stepping performance, in a group of elderly fallers that performed perturbation exercises. Melzer and Oddsson [14], Mansfield et al. [13] and Rogers et al. [44] also found that specific step training that includes perturbation of balance improves stepping abilities in older adults. Pai et al. [12] have shown reductions in falls and balance loss following a repeated-slip during walking exposure. The same group had further showed that those gains could be retained for 6 months [18] and cut older adults’ annual risk of falls by 50 % especially among elderlies who had history of falls [19]. Our results and the results above suggests that specificity-of-training principle is a major factor in achieving treatment goals and therapists need to tailor balance perturbation training programs to target functional aspects of balance control such as the ability to step rapidly [45].

It is still unknown how the improvement seen in balance and stepping carries over to real-life falls. It may be that carryover to fear of falls and physical performance as measure with POMA did exist but was not detected by these outcomes measures due to insufficient sample size or due to ceiling effects, subjects were close to score the highest score in both measures. We also did not find significant carryover effects on self-reported and performance based function. This suggests that function is not derived only by the ability to perform balance tasks, but is influenced by environmental and behavioral factors. Most perturbation training programs did not measure self-reported physical function [16–19, 40, 44]. However other studies found improvement in self-reported function [14] and fear of falls [15]. This two studies trained balance in a group setting adding also behavioral factors. This may suggest that physical intervention per-se without behavioral intervention cannot change the levels of physical function. The fact that Performance based measure are weakly associated with self-reported mobility in healthy elderly persons (*r* = 0.21–0.29) [46] support this notion.

This study has several limitations. First, the data came from a fairly small sample of independent older adults thus the results cannot be generalized to frail or institutionalized elderly persons. Second, the training program was done on a treadmill and not on leveled ground. Treadmill walking is somewhat different then over ground walking and might pose different demands on the trainee. However, it has been showed that fall-resisting skills acquired from such training can be transferred to over-ground walking [47]. Third, to provide stronger evidence of the clinical efficacy of this training procedure, future studies should compare the current intervention with an alternative treatment such as Tai Chi, or strength training as this was found beneficial in a recent review [48]. Also there is a need to determine whether this type of training improves the ability to cope with falls that resulted from slips and trips only, or can be transferred to any kind of loss of balance. Fourth, further research is needed in order to determine whether randomly ordered perturbations exercises (Random practice) would render better results than repeating the same perturbation exercises (blocked practice) in terms of acquisition, adaptation and retention.

## Conclusion

Participation in a balance training program that includes unexpected perturbation of balance during treadmill walking reduced the risk factors for falls as presented by biomechanical markers that have been shown in the past to identify fallers and predict falls. However levels of physical functioning were not improved, this suggests that preventing sedentary lifestyle for the elderly require additional behavioral intervention.
